# Supplementary figures and images for: Analysis of mouse lens morphological and proteomic abnormalities following depletion of βB3-crystallin
Source: bioRxiv. 2024 Dec 30:2024.12.30.630781. Preprint. [Version 1] doi: 10.1101/2024.12.30.630781 (PMC11722438; doi:10.1101/2024.12.30.630781)

Supplementary Fig. 1

a.

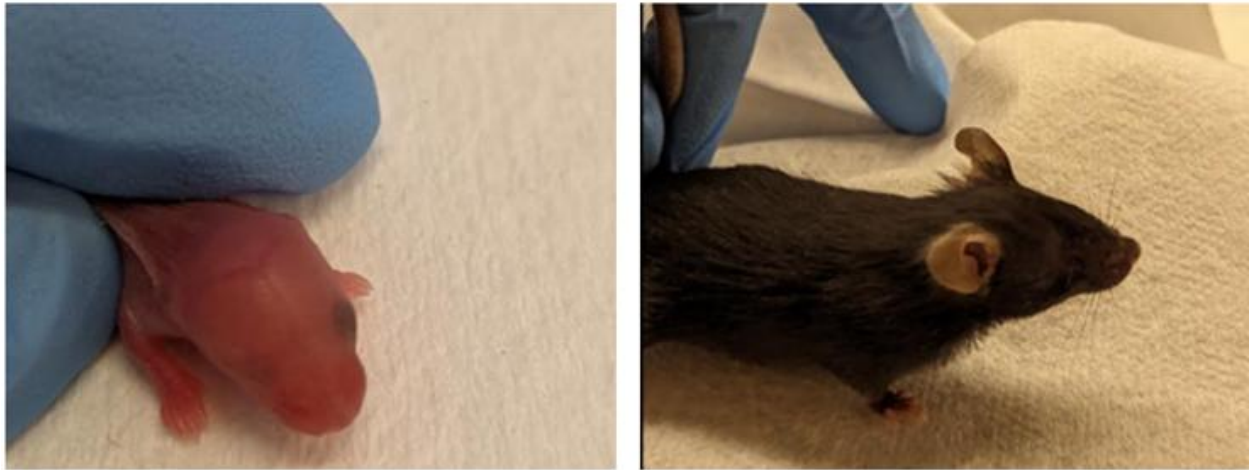

b.

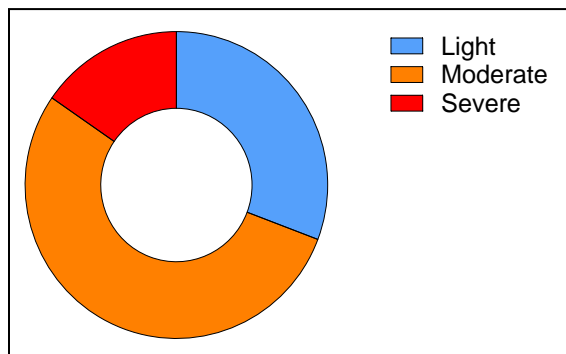

Supplementary Fig. 2

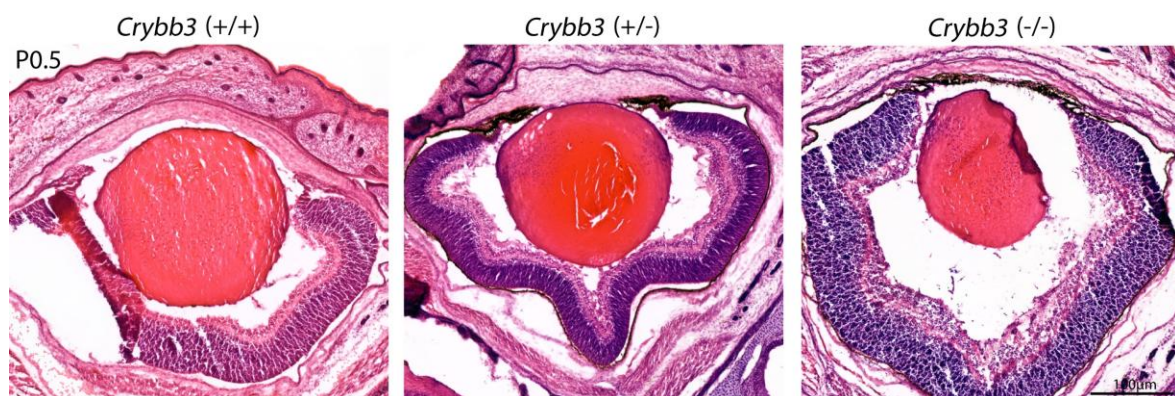

Supplement: Supplement 1 — Supplementary Figure S1: Phenotype variability in KO mice lacking Crybb3 promoter sequence. A. P0 and 3-month-old mice displaying complete left eye absence. B. KO mice display a variable lens morphology spectrum from light to severe (eye absence) phenotype. Most of the animals analyzed display moderate lens morphological abnormalities. N = 15 KO analyzed animals from independent litters at P0 stage compared to WT. Parameters measured: lens area relative to control; organelle-free zone absence. Supplementary Figure S2: WT, heterozygous and knockout newborn lenses morphology. [file media-1.pdf]
